# NO_2_ Sensing Capability of Pt–Au–SnO_2_ Composite Nanoceramics at Room Temperature

**DOI:** 10.3390/molecules28041759

**Published:** 2023-02-13

**Authors:** Jiannan Song, Zhongtang Xu, Menghan Wu, Xilai Lu, Zhiqiao Yan, Feng Chen, Wanping Chen

**Affiliations:** 1 Key Laboratory of Artificial Micro- and Nano-Structures of Ministry of Education, School of Physics and Technology, Wuhan University, Wuhan 430072, China; 2 Key Laboratory of Applied Superconductivity, Institute of Electrical Engineering, Chinese Academy of Sciences, Beijing 100190, China; 3 Guangdong Provincial Key Laboratory of Metal Toughening Technology and Application, Institute of New Materials, Guangdong Academy of Sciences, Guangzhou 510650, China

**Keywords:** NO_2_, Pt–Au–SnO_2_, sensor, room temperature

## Abstract

Composite ceramics of metal oxides and noble metals have received much attention for sensing reducing gases at room temperature. Presently, composite ceramics of SnO_2_ and noble metals have been prepared and investigated for sensing oxidizing NO_2_ at room temperature. While dramatic increases in resistance were observed for both 1 wt% Pt–SnO_2_ and 5 wt% Au–SnO_2_ composite nanoceramics after being exposed to NO_2_ at room temperature, the largest increase in resistance was observed for 1 wt% Pt–5 wt% –Au–SnO_2_ composite nanoceramics among the three composites. The response to 0.5 ppm NO_2_-–20% O_2_–N_2_ was as high as 875 at room temperature, with a response time of 2566 s and a recovery time of 450 s in the air of 50% relative humidity (RH). Further investigation revealed that water molecules in the air are essential for recovering the resistance of Pt–Au–SnO_2_ composite nanoceramics. A room temperature NO_2_-sensing mechanism has been established, in which NO_2_ molecules are catalyzed by Pt–Au to be chemisorbed on SnO_2_ at room temperature, and desorbed from SnO_2_ by the attraction of water molecules in the air. These results suggest that composite ceramics of metal oxides and noble metals should be promising for room temperature sensing, not only reducing gases, but also oxidizing gases.

## 1. Introduction

Due to more and more fuel combustion in places such as thermal power plants and automobiles, NO_2_ in the air has been increasing quickly in recent years [1,2]. NO_2_ is not only directly harmful to human health, but also results in soil contamination through the formation of acid rain [3,4,5,6]. With the advantages of good sensitivity and selectivity, electrochemical gas sensors have been most widely used for NO_2_ detection [7,8]. However, their relatively short service life [8], susceptibility to environmental interference, and poor stability, have caused much inconvenience for NO_2_ detection. Other NO_2_ gas sensors, with good stability, long service life, and low price, are highly expected.

There have been extensive investigations devoted to developing NO_2_ gas sensors based on metal oxides [9,10,11,12,13,14], and many of them have been focused on developing room-temperature metal oxide NO_2_ gas sensors. Room temperature operation is not only important for low power consumption, but also for miniaturization. Several research groups have made some impressive progresses by adopting nanostructured metal oxides. For example, Zhang et al. have prepared SnO_2_–ZnO with a layered nanostructure, which showed responses to ppb level NO_2_ at 150 °C [15]. Bang et al. have synthesized SnS-nanoparticle-functionalized SnO_2_ nanowires, which could detect 2 ppm NO_2_ at 100 °C [16]. Han et al. have synthesized brick-like In_2_O_3_ nanomaterials, which exhibited a 402 response to 500 ppm NO_2_ at 50 °C [17]. Liu et al. have synthesized hollow SnO_2_–SnS_2_ nanostructures, which achieved room-temperature NO_2_ detection by visible light irradiation [18]. Pham et al. have fabricated MoS_2_ single-layer films, which could detect NO_2_ at room temperature with red-light irradiation [19]. Although the introduction of light irradiation has brought the operating temperature down to room temperature, it will cause some inconvenience in itself. It has to be pointed out that these low-dimensional-nanostructured materials that have been investigated are of poor mechanical strength and are relatively complicated in composition and structure, which will be unfavorable for practical applications. Many more explorations are highly desirable to develop relatively simple and robust room-temperature NO_2_ sensors based on metal oxides.

As a matter of fact, to develop room-temperature gas sensors based on metal oxides with a relatively high mechanical strength, two different strategies have already emerged. In the first strategy, a porous nanosolid (PNS) is prepared from metal oxide nanoparticles through a solvothermal hot press (SHP) [20]. PNS is considered an intermediate state between nanoparticles and nanoceramics [21,22], and with both high reactivity of nanoparticles and strength of nanoceramics: some impressive room temperature metal-oxide gas sensors have been fabricated from PNSs [23,24]. In the second strategy, the catalytic effect of noble metals (e.g., Pt and Pd) is utilized to achieve room-temperature gas sensing. Highly remarkable room-temperature gas sensing capabilities have been observed in many composite ceramics of noble metals and metal-oxide semiconductors (e.g., TiO_2_, WO_3_, Nb_2_O_5_, SnO_2_ and ZnO), which were prepared through traditional pressing and sintering [25,26,27,28,29,30]. It is worthy to note that for composites of Pt with micron-sized WO_3_ and SnO_2_ agglomerate powder [26,27], surprisingly strong room-temperature responses to hydrogen and an extraordinarily high moisture resistance have been observed, which is especially important for practical room-temperature gas sensing applications. Obviously, this strategy is highly appealing for developing room-temperature metal-oxide gas sensors, with promising practical application potentials. However, it has to be pointed out that bulk ceramics prepared through this strategy can only sense reducing gases of H_2_ and CO at room temperature at present, in which noble metals, such as Pt and Pd, promote H_2_ and CO to react with the oxygen chemisorbed on the metal oxides, and even be chemisorbed on the metal oxides at room temperature [31,32,33,34,35]. There have been no reports on preparing bulk materials capable of sensing oxidizing gases, including NO_2_, at room temperature through this strategy up to date.

In a previous investigation, the resistance of Pt–SnO_2_ nanoparticles was found to increase dramatically with increasing Pt content, which clearly indicates that Pt can promote the chemisorption of oxygen molecules on SnO_2_ at room temperature [36]. In another investigation, Pd was also found to be able to promote oxygen chemisorption on SnO_2_ in Pd–SnO_2_ nanoparticles at room temperature through XPS analyses [29]. It is well known that O_2_ is a typically oxidizing gas. These facts suggest that noble metals, such as Pt and Pd, may also be able to promote some oxidizing gases to be chemisorbed on some metal oxides at room temperature, and in turn, their composites, with these metal oxides, should be able to show responses to the oxidizing gases at room temperature. Thus, this indicates that bulk composites capable of sensing oxidizing gases at room temperature should be possibly obtained through the second strategy. Presently, we have adopted this strategy to prepare Pt–SnO_2_ composite nanoceramics through pressing and sintering, which were indeed found to show strong responses to NO_2_ at room temperature. More interestingly, the room-temperature NO_2_ sensing characteristics were further dramatically improved through the introduction of Au to the composites, and a rather remarkable room-temperature NO_2_ sensing capability has been observed for Pt–Au–SnO_2_ composite nanoceramics. Some further studies have been conducted, which show that water molecules in the air play a vital role for those samples to recover their resistance in the air after being exposed to NO_2_. It is proposed that NO_2_ molecules are catalyzed by Pt–Au to be chemisorbed on SnO_2_ at room temperature, and are removed from SnO_2_ by the attraction of water molecules in the air. These results clearly demonstrate that metal-oxide bulk materials capable of sensing oxidizing gases at room temperature can be prepared through pressing and sintering. However, more investigation regarding this is highly desirable.

## 2. Results and Discussion

### 2.1. Phase and Morphological Investigations

**Figure 1:**
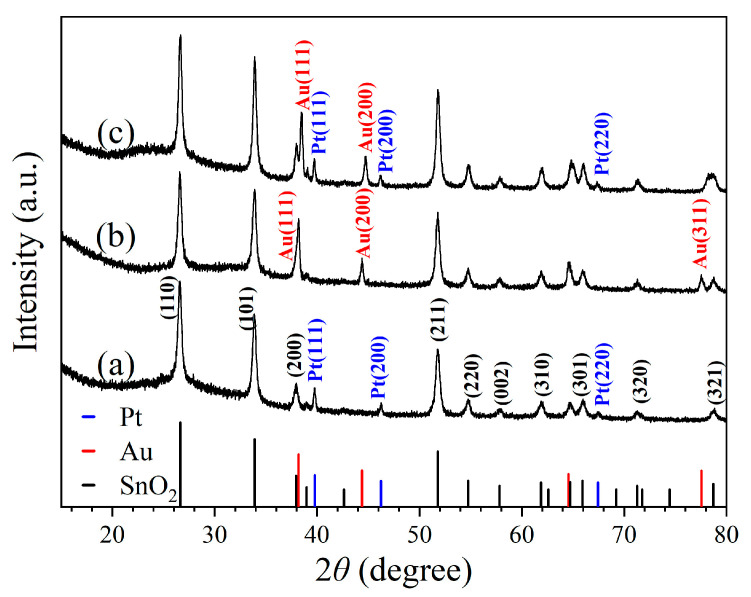
X-ray diffraction patterns taken for samples of (**a**) 1 wt% Pt–SnO_2_, (**b**) 5 wt% Au–SnO_2_, and (**c**) 1 wt% Pt–5 wt% Au–SnO_2_, after being sintered at 950 °C for 2 h in the air.

Figure 1 shows the X-ray diffraction (XRD) patterns obtained for three kinds of nanoceramics, of which the nominal compositions/sintering temperatures are 1 wt% Pt–SnO_2_/950 °C, 5 wt% Au–SnO_2_/950 °C and 1 wt% Pt–5 wt% Au–SnO_2_/950 °C, respectively. As shown in Figure 1a, for 1 wt% Pt–SnO_2_, three diffraction peaks can be clearly identified as (111), (200) and (220) planes of cubic Pt, according to JCPDS 04-0802. All other diffraction peaks can be identified as planes of rutile SnO_2_, according to CPDS 78-1063. Obviously, this sample was a composite of the cubic Pt and rutile SnO_2_. Figure 1b illustrates the XRD diffraction pattern of 5 wt% Au–SnO_2_. Similarly, all the diffraction peaks can be identified as planes of rutile SnO_2_ and cubic Au (JCPDS 65-2870) respectively, indicating a composite of Au and SnO_2_. Figure 1c presents the XRD pattern of 1 wt% Pt–5 wt% Au–SnO_2_. Beside the peaks from the rutile SnO_2_, some peaks from both the cubic Au and Pt can be observed, which indicates that Pt and Au existed as separate phases in this sample. Therefore, this sample of 1 wt% Pt–5 wt% Au–SnO_2_ was a composite of the cubic Pt, cubic Au, and rutile SnO_2_. As a matter of fact, both Pt and Au are highly stable noble metals, and it is reasonable that they can form composites with SnO_2_ through high-temperature sintering.

**Figure 2:**
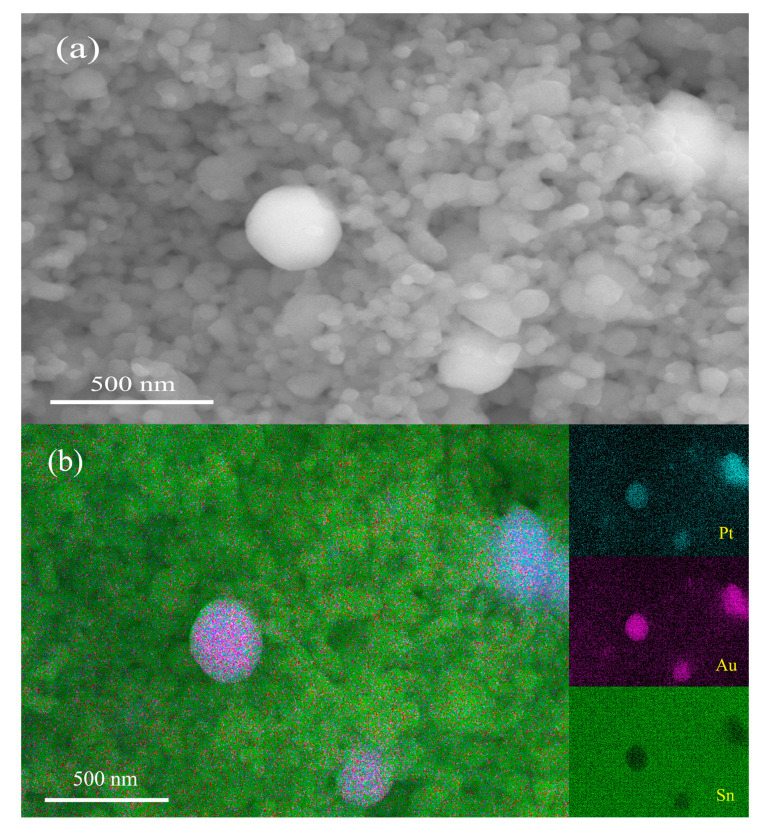
(**a**) SEM micrograph and (**b**) EDS analysis taken for a fractured surface of a sample of 1 wt% Pt–5 wt% Au–SnO_2_, after being sintered at 950 °C for 2 h in the air.

As pointed out in some previous papers, the SnO_2_ nanoparticles used in this study exhibit a very unique sintering behavior, and pellets prepared from them showed no sintering shrinkage, even after being sintered at 1200 °C [29]. Accordingly, all the samples prepared in this study showed no noticeable sintering shrinkage either. Figure 2a shows an SEM micrograph obtained for a fractured surface of a sample of 1 wt% Pt–5 wt% Au–SnO_2_ nanoceramics sintered at 950 °C for 2 h in the air. Firstly, some nanopores can be clearly observed in the micrograph, which should be helpful for gas sensing. It can be observed that most grains are about 70 nm in diameter, while a few much larger grains, around 300 nm in diameter, can also be observed. According to the EDS analysis shown in Figure 2b, those smaller grains should be SnO_2_ nanograins, and they must have experienced no obvious grain growth in the sintering, as they were quite similar to SnO_2_ nanoparticles in size. The much larger grains were Pt and Au grains, respectively. The Au powder and Pt powder were marked as <500 nm and <1 μm, respectively. Thus, these large grains must have come from their starting materials. According to two very recent papers, large Pt grains are important for room-temperature CO-sensitive and H_2_-sensitive Pt–SnO_2_ composite nanoceramics to achieve a high, long-term stability [37,38]. As a matter of fact, these two commercial Au and Pt powders, with relatively large particles, had been intentionally chosen as starting materials in this study.

### 2.2. Room-Temperature NO_2_-Sensing Measurement

**Figure 3:**
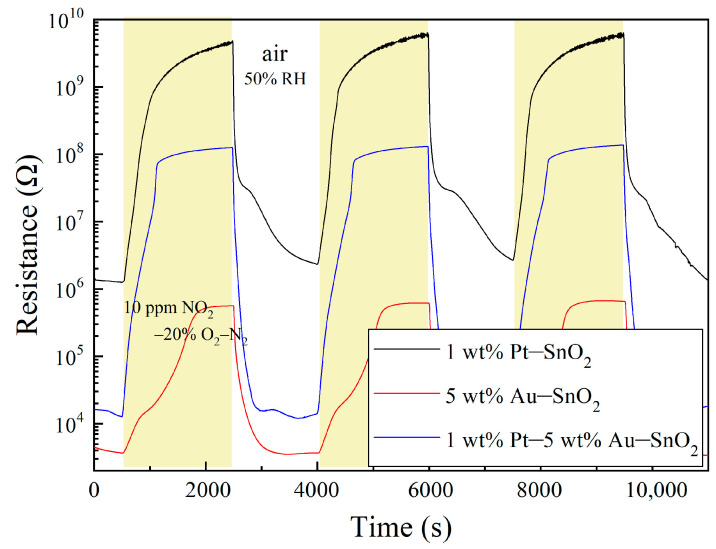
Room-temperature response to 10 ppm NO_2_ in 20% O_2_–N_2_ and recovery in the air of 50% RH for three samples with compositions of 1 wt% Pt–SnO_2_, 5 wt% Au–SnO_2_ and 1 wt% Pt–5 wt% Au–SnO_2_, respectively.

Pt–SnO_2_ composite nanoceramics have been found to show strong responses to the reducing gases of H_2_ and CO at room temperature, which is characterized by a dramatic decrease in their resistance upon being exposed to the gases [29,36]. According to our knowledge, however, there have been no reports on the room-temperature responses of the Pt–SnO_2_ composite nanoceramics to any oxidizing gases up to date. It is, thus, very surprising to see that the Pt–SnO_2_ composite nanoceramics prepared in this study exhibited an extraordinarily strong response to 10 ppm NO_2_–20% O_2_–N_2_ at room temperature, as shown in Figure 3. For the sensing of oxidizing gases, the response *S* is usually defined as *S* = *R*_g_/*R*_a_, where *R*_g_ and *R*_a_ represent the resistance of the sensor in the target gas and in the air, respectively, and response (recovery) time is defined as the time taken by the sensor to reach 90% of the total resistance change after the introduction (discontinuation) of the gas for testing [15]. According to this definition, this sample of 1 wt% Pt–SnO_2_ had a room-temperature response of 923 with a response time of 614 s and a recovery time of 1350 s to 10 ppm NO_2_–20% O_2_–N_2_. Such a room temperature response to NO_2_ is highly outstanding when compared with those of the newly reported metal oxides in the literature.

For reference, other kinds of composites had also been prepared and investigated for room-temperature NO_2_ sensing. A very interesting result was observed for a sample of 5 wt% Au–SnO_2_, sintered at 950 °C for 2 h in the air, as shown in Figure 3. Firstly, it can be seen that this sample had a very low resistance in the air, which indicates that Au is much less effective than Pt in promoting the chemisorption of O_2_ molecules on SnO_2_ at room temperature. Secondly, this sample also showed a strong response to 10 ppm NO_2_–20% O_2_–N_2_ at room temperature, with a response of 132, a response time of 1279 s and a recovery of 672 s. It seems that Au has a stronger catalytic effect on NO_2_ molecules than O_2_ molecules, with regards to their chemisorption on SnO_2_ at room temperature.

For the sample of 1 wt% Pt–SnO_2_, the response was very attractive, but its resistance to NO_2_ was too high to ensure a stable measurement. The signal was a little unstable at the top of the curve. When regarding the sample of 5 wt% Au–SnO_2_, it had a much smaller resistance in NO_2_, but its response to NO_2_ was also much weaker than that of the former sample. Clearly, these two samples had some rather complementary advantages and disadvantages. Therefore, we had prepared composites of 1 wt% Pt–5 wt% Au–SnO_2_, and the result was very surprising. As shown in Figure 3, impressive room-temperature NO_2_-sensing properties are observed for a sample of 1 wt% Pt–5 wt% Au–SnO_2_. First, this sample had a much lower resistance in the air than the sample of 1 wt% Pt–SnO_2_, which indicates that the room temperature chemisorption of O_2_ molecules on SnO_2_ in the presence of both Pt and Au is much different from that in the presence of Pt alone. Secondly, this sample showed an extraordinarily strong response to NO_2_ at room temperature, with a response of 6031, a response time of 591 s, and a recovery time of 430 s to 10 ppm NO_2_–20% O_2_–N_2_. Due to its rather small resistance in the air, the signal was quite stable in NO_2_, even with such a strong response. It is worthy to note that this sample had the strongest response to NO_2_ at room temperature among the three samples, which is actually very difficult to understand.

**Figure 4:**
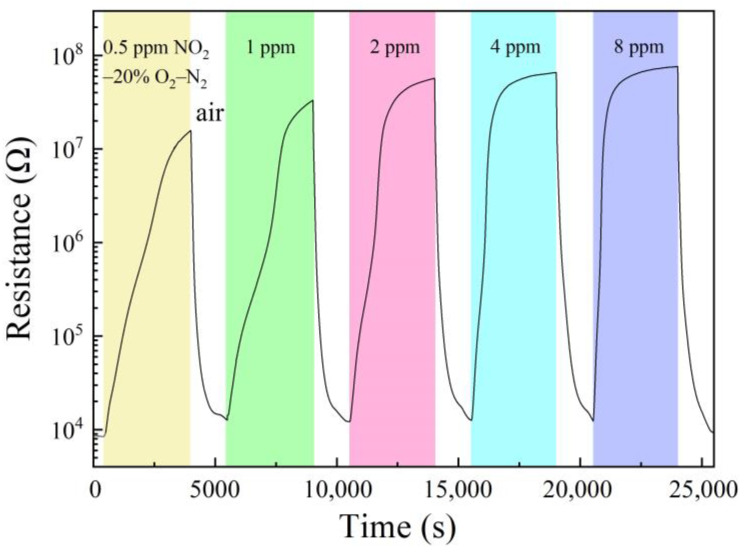
Resistance response to a series of concentrations of NO_2_ in 20% O_2_–N_2_ at room temperature and recovery in the air of 50% RH, for a sample of 1 wt% Pt–5 wt%Au–SnO_2_ sintered at 950 °C for 2 h the in air.

The sample of 1 wt% Pt–5 wt% Au–SnO_2_ had been exposed to NO_2_ in a series of concentrations at room temperature and the results are shown in Figure 4. Its room-temperature response to 0.5 ppm NO_2_–20% O_2_–N_2_ was 875, with a response time of 2566 s, and a recovery time of 450 s. With increasing NO_2_ concentration, both the response and the response speed increased steadily. To 8 ppm NO_2_–20% O_2_–N_2_, the response was increased to 4300 with a much smaller response time of 924 s and a recovery time of 440 s. It is interesting to compare this sample with some representative NO_2_-sensitive nanomaterials newly reported in the literature, as shown in Table 1. It can be clearly seen that our sample had a much higher room-temperature response to a low concentration NO_2_ than all these low-dimensional nanomaterials, and as a bulk material, our sample should also have a much higher mechanical strength. Obviously, these results indicate that the composite nanoceramics of 1 wt% Pt–5 wt% Au–SnO_2_ prepared in this study should be highly attractive for room-temperature NO_2_ sensing.

### 2.3. Mechanism Study on Room-Temperature NO_2_ Sensing Characteristics

**Figure 5:**
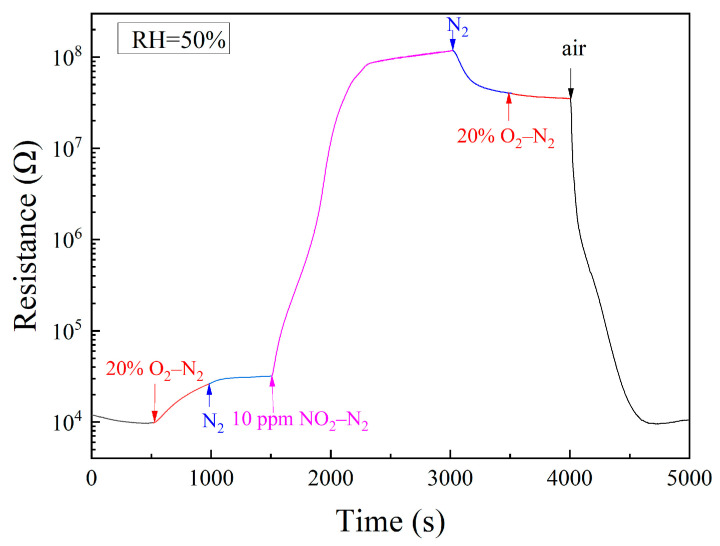
Room-temperature resistance responses to a sequence of atmospheres: air, 20% O_2_–N_2_, N_2_, 10 ppm NO_2_–N_2_, N_2_, 20% O_2_–N_2_, and air for a sample of 1 wt% Pt–5 wt% Au–SnO_2_ sintered at 950 °C.

As an oxidizing gas, NO_2_ increases the resistance of n-type semiconductors through its chemisorption, which is the same as what oxygen does. It is meaningful to identify the influence of NO_2_ from that of oxygen in NO_2_ sensing. For this purpose, we had exposed a sample of 1 wt% Pt–5 wt% Au–SnO_2_ to a series of specifically designated atmospheres at room temperature, as shown in Figure 5. When the atmosphere was first changed from air to 20% O_2_–N_2_, the resistance slowly increased with time, which actually indicates a drying effect of the flowing gas of 20% O_2_–N_2_ in the sample. Those oxygen molecules chemisorbed on SnO_2_ must be very stable, so there was no turning point when the atmosphere was changed from 20% O_2_–N_2_ to N_2_. Upon being exposed to 10 ppm NO_2_–N_2_, a steep increase was observed when the atmosphere was changed from N_2_ to 10 ppm NO_2_–N_2_. The resistance was finally increased by more than three orders of magnitude, which demonstrates a strong chemisorption of NO_2_ on SnO_2_. When the surrounding atmosphere was changed from 10 ppm NO_2_–N_2_ to N_2_, the resistance only decreased very slowly with time, which further confirms a strong chemisorption of NO_2_ on SnO_2_. No turning point appeared when the atmosphere was changed from N_2_ to 20% O_2_–N_2_. It is worth mentioning that when the atmosphere was changed from 20% O_2_–N_2_ to N_2_ and from N_2_ to 20% O_2_–N_2_, respectively, only the oxygen content was changed. The absence of the turning point for oxygen content change in the resistance versus the time curve indicates a stable oxygen chemisorption on SnO_2_. Considering that both NO_2_ and O_2_ are strongly chemisorbed on SnO_2_ at room temperature, it was highly surprising to see that the resistance was sharply decreased when the surrounding atmosphere was changed from 20% O_2_–N_2_ to the air of 50% RH, as shown in Figure 5. Given the difference between these two atmospheres, water molecules in the air had obviously played the vital role in this resistance decrease.

**Figure 6:**
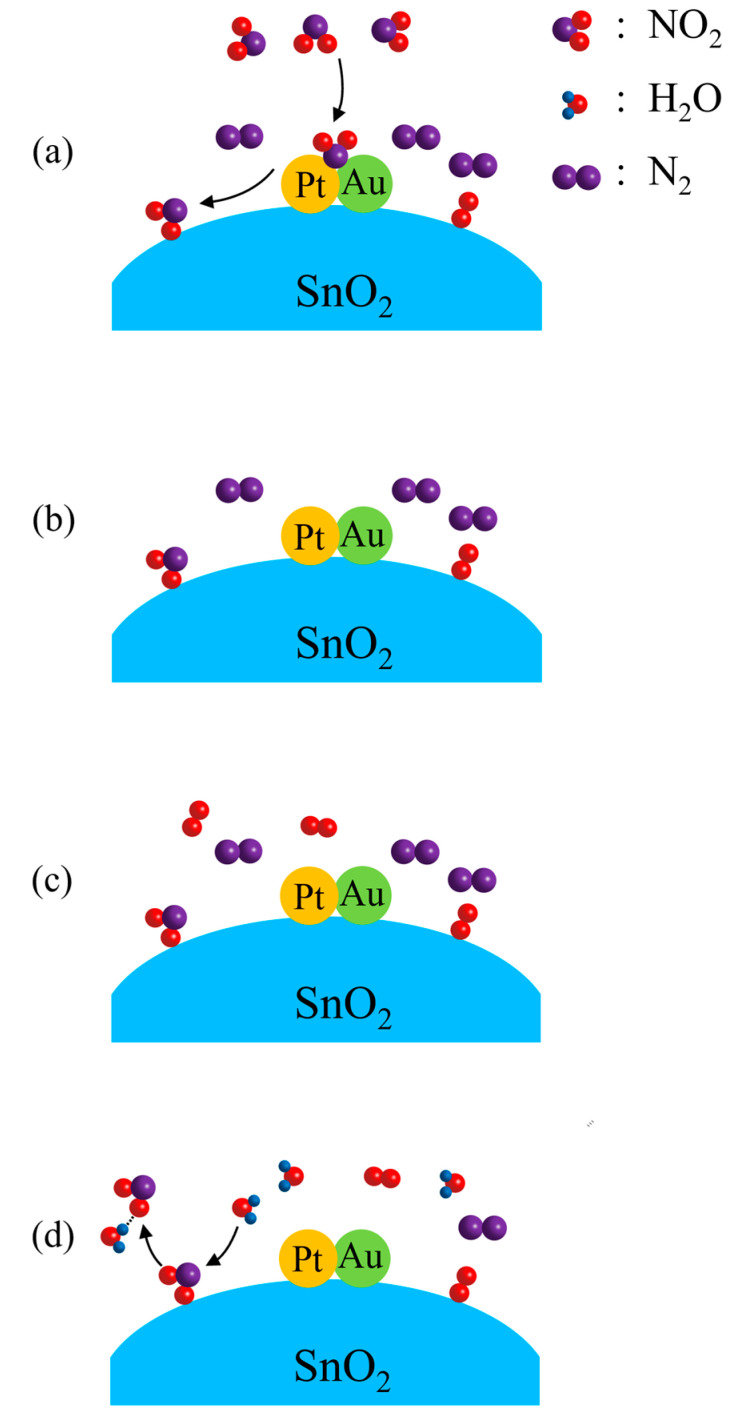
Schematic illustrations for Pt–Au–SnO_2_ composite nanoceramics at room temperature in: (**a**) 10 ppm NO_2_–N_2_, where NO_2_ molecules are chemisorbed on SnO_2_ catalyzed by Pt–Au; (**b**) N_2_, where NO_2_ molecules are stably chemisorbed on SnO_2_; (**c**) 20% O_2_–N_2_, where no more O_2_ molecules are chemisorbed on SnO_2_, as those O_2_ molecules chemisorbed earlier have remained there; (**d**) air of 50% RH, where NO_2_ molecules are desorbed from SnO_2_ by the attraction of water molecules in the air.

The chemisorption of NO_2_ on SnO_2_ at room temperature in 10 ppm NO_2_–N_2_ can be expressed as [43]:

(1) $$NO_{2(\mathrm{gas})}+e^{\prime }\to NO_{2(\mathrm{adsorbed})}^{-}$$

in which Pt–Au must have acted as the catalyst for the reaction, as shown in Figure 6a. As an oxidizing gas, NO_2_ molecules accept electrons from SnO_2_ when they are chemisorbed on SnO_2_. This explains the steep increase in resistance when the sample is exposed to 10 ppm NO_2_–N_2_. When the surrounding atmosphere is changed from 10 ppm NO_2_–N_2_ to N_2_, the chemisorption of NO_2_ is so stable that most NO_2_ molecules will not leave SnO_2_ in the surrounding N_2_, as shown in Figure 6b. In this way, only a slight decrease in resistance can be observed. When the surrounding atmosphere is changed from N_2_ to 20% O_2_–N_2_, no more O_2_ molecules can be chemisorbed on SnO_2_, as those O_2_ molecules chemisorbed on SnO_2_ earlier have remained there, as shown in Figure 6c. Thus, no resistance change can be observed for this atmosphere change. H_2_O and NO_2_ molecules are able to form NO_2_–H_2_O clusters through the hydrogen bonds between them [44]. When the ambient atmosphere is changed from 20% O_2_–N_2_ to the air of 50% relative humidity, NO_2_ molecules are desorbed from SnO_2_ by the attraction of H_2_O molecules, as shown in Figure 6d. This desorption of NO_2_ can be expressed as:

(2) $$NO_{2(\mathrm{adsorbed})}^{-}+H_{2}O\to H_{2}O-NO_{2}+e^{\prime }$$

The electrons captured by the NO_2_ molecules are returned to SnO_2_, so the resistance is greatly decreased when air with H_2_O molecules is introduced. This forms a sharp contrast with the resistance recovery in the air for n-type metal oxides after being exposed to reducing gases at room temperature, in which oxygen molecules in the air are chemisorbed on the metal oxides and the resistance is increased.

## 3. Materials and Methods

### 3.1. Material Preparation

SnO_2_ nanoparticles (70 nm, 99.99%), a commercial Au powder (<500 nm, 99.9%), and a commercial Pt powder (<1 μm, 99.9%) from Aladdin, Shanghai, China, were used as the starting materials. According to designated ratios, these particles were mixed in deionized water and magnetically stirred. For every suspension, magnetic stirring was performed for 10 h to ensure homogeneous mixing, and then the suspension was dried in an oven at 120 °C for 2 h. After grinding, the dried powders were homogenized with deionized water as a binder, and pressed using a hydraulic press at 3 MPa to form pellets with a diameter of 10 mm and a thickness of 1 mm. The pellets were sintered at 850–1050 °C for 2 h in air. For gas-sensing measurement, a pair of rectangular gold electrodes was formed on a major surface of a sample, through direct-current (DC) magnetron sputtering.

### 3.2. NO_2_-Sensing Measurement

A commercial gas-sensing measurement system (GRMS-215, Partulab Com., Wuhan, China), which has been described in detail in previous papers [31,45], was used for the tests, mainly composed of a 350 mL quartz-sealed chamber and a computer for recording data. The sealed chamber contained four inlet tubes and one exhaust tube. Four inlet tubes were connected to N_2_ (99.999%, Zhongxinruiyuan Gas, Wuhan, China), O_2_ (99.999%, Zhongxinruiyuan Gas, Wuhan, China), 15 ppm NO_2_–N_2_ (97.0%, Foshan Kodi Gas, Foshan, China), and the air, to realize designated internal atmospheres. A flow controller was used to control the gas inflow in every pipe and ensure a stable gas flow. During the response stage, NO_2_, N_2_, and O_2_ entered the chamber at specific rates through three tubes, and the total rate was maintained at 300 mL/min. During the recovery stage, ambient air was pumped into the chamber at a rate of 10 L/min. During most measurements, the room temperature was kept at 25 °C and the RH in the air remained around 50%.

### 3.3. Material Characterization

Crystal structures of the prepared samples were investigated by powder X-ray diffraction (BRUKER AXS D8 ADVANCE, Bruker Daltonics, Bremen, Germany), using Cu K_α_ radiation. Morphologies and microstructures of the nanoceramics were characterized by scanning electron microscopy (SEM; SIRION, FEI, Eindhoven, the Netherlands). The distribution of elements was studied by energy-dispersive spectroscopy (EDS, ZEISS Corporation, Jena, Germany), using the OXFORD Aztec 250 instrument (Oxford Instruments, Oxford, UK).

## 4. Conclusions

Composite nanoceramics have been prepared through the pressing and sintering of Pt, Au, and SnO_2_ nanoparticles. For samples of 1 wt% Pt–SnO_2_, the resistance was greatly increased after being exposed to NO_2_ in synthetic air at room temperature. For samples of 5 wt% Au–SnO_2_, the resistance was unusually small in the air, and was dramatically increased by NO_2_ in synthetic air at room temperature. Samples of 1 wt% Pt–5 wt% Au–SnO_2_ had the strongest response to NO_2_ at room temperature, with a response of 875 to 0.5 ppm NO_2_–20% O_2_–N_2_, a response time of 2566 s, and a recovery time of 450 s in the air of 50% RH. Such a room-temperature NO_2_-sensing capability is highly remarkable among those reported in the literature. According to the mechanism study results, it is proposed that NO_2_ molecules are chemisorbed on SnO_2_ under the catalytic effect of Pt–Au at room temperature, and water molecules in the air have a tendency to desorb NO_2_ molecules from SnO_2_ through attraction. More studies on composite ceramics of metal oxides and noble metals for sensing oxidizing gases at room temperature are highly desirable.

## Figures and Tables

**Table 1:** Performances of representative low-temperature NO_2_-sensing materials.

Sensing Materials	Response/NO_2_ Concentration	Measuring Temperature	Ref.
Sn-doped In_2_O_3_ nanofibers	44.6/1 ppm	90 °C	[9]
Pt–SnO_2_ porous spheres	2/0.5 ppm	80 °C	[39]
Pt–Bi_2_O_3_–SnO_2_ nanowires	27.7/1 ppm	50 °C	[40]
SnO_2_ NP–RGO hybrids	3.8/1 ppm	25 °C	[41]
2D SnS_2_	301/1 ppm	25 °C	[42]
Pt–Au–SnO_2_ nanoceramics	875/0.5 ppm	25 °C	This work

## Data Availability

Data are available on request due to restrictions privacy.

## Sample Availability

Not available.
